# Salinity-induced changes in plastoquinone pool redox state in halophytic *Mesembryanthemum crystallinum* L.

**DOI:** 10.1038/s41598-023-38194-7

**Published:** 2023-07-10

**Authors:** Maria Pilarska, Ewa Niewiadomska, Jerzy Kruk

**Affiliations:** 1 The Franciszek Górski Institute of Plant Physiology, Polish Academy of Sciences, Niezapominajek 21, 30-239 Kraków, Poland; 2 Department of Plant Physiology and Biochemistry, Faculty of Biochemistry, Biophysics and Biotechnology, Jagiellonian University, Gronostajowa 7, 30-387 Kraków, Poland

**Keywords:** Photosynthesis, Plant stress responses

## Abstract

We have analyzed the effect of salinity on photosystem II (PSII) photochemistry and plastoquinone (PQ) pool in halophytic *Mesembryanthemum crystallinum* plants. Under prolonged salinity conditions (7 or 10 days of 0.4 M NaCl treatment) we noted an enlarged pool of open PSII reaction centers and increased energy conservation efficiency, as envisaged by parameters of the fast and slow kinetics of chlorophyll *a* fluorescence. Measurements of oxygen evolution*,* using 2,6-dichloro-1,4-benzoquinone as an electron acceptor, showed stimulation of the PSII activity due to salinity. In salt-acclimated plants (10 days of NaCl treatment), the improved PSII performance was associated with an increase in the size of the photochemically active PQ pool and the extent of its reduction. This was accompanied by a rise in the NADP^+^/NADPH ratio. The presented data suggest that a redistribution of PQ molecules between photochemically active and non-active fractions and a change of the redox state of the photochemically active PQ pool indicate and regulate the acclimation of the photosynthetic apparatus to salinity.

## Introduction

In plant cells, salinity disturbs ion homeostasis and decreases leaf water potential and turgor pressure. Such a situation leads to ionic and osmotic stress, which enhances reactive oxygen species (ROS) production^1, 2^. To counteract that, plants modulate the antioxidant system and intensify the biosynthesis of osmoprotectants and phytohormones, which increases the energy requirements of the cells^1^. The environmental changes affect also the functioning of chloroplasts, which are sensitive to external factors. Salinity perturbs the chlorophyll biosynthesis, integrity of photosynthetic apparatus, and reduces the activity of Calvin cycle enzymes^3^. Such conditions can disrupt the balance between the photosynthetic electron transport (PET) chain and carbon assimilation, and may lead to oxidative stress^4^. Therefore, acclimation to salinity requires adjusting the photosynthesis functioning along with metabolism reprogramming.

Numerous data indicate, that changes of the redox state of PET components, including plastoquinone (PQ), appear to be crucial signals triggering stress responses^5^. The photochemically active PQ (hereafter PQ_PA_) pool located in thylakoids is reduced by photosystem II (PSII) and oxidized by cytochrome b_6_f complex^6^. However, the PQ_PA_ redox state is affected by various light-dependent and independent electron fluxes, including cyclic electron transport (CET)^6, 7^, chlororespiration^8, 9^ and ‘pseudocyclic’ electron transfer^10^. In addition, PQ and its reduced form, plastoquinol (PQH_2_), are also found in plastoglobuli and in the chloroplast envelope membrane, where they are photochemically non-active^6, 11^.

PQ is involved in miscellaneous physiological processes, and is an important element of plant stress acclimation. PQ-redox driven signal(s) regulate the expression of PSII and photosystem I (PSI) proteins, superoxide dismutases (SODs), ascorbate peroxidase (APX), and others^12–14^. PQ can be a source of hydrogen peroxide^15^, but on the other hand, it has also an antioxidant function, as in a reduced state it scavenges singlet oxygen, hydrogen peroxide and superoxide^16, 17^. The redox state of the PQ_PA_ pool is also involved in state transitions through the regulation of LHCII phosphorylation by STN7 kinase^18^, and plays a crucial role in the biosynthesis of carotenoids^19^, plastochromanol and phylloquinone^20^.

Halophytes, due to their ability to grow in saline soils, represent a useful model to study complex physiological and genetic mechanisms of salinity stress tolerance^21^. One of the most intensively studied annual facultative halophyte is *Mesembryanthemum crystallinum*, the common ice plant, where salinity induces a transition from C_3_ photosynthesis to Crassulacean acid metabolism (CAM)^22^. The dark CO_2_ uptake and its fixation in the Calvin cycle during the day is a strategy to maintain photosynthesis despite closed stomata and greatly enhances water usage efficiency^23^. In ice plant, however, CAM induction is not an obligatory mechanism for basic salinity resistance^24^. Also, other strategies have been described, including the synthesis of osmoprotectants, Na^+^ accumulation in specialized cell trichomes^25^, and the induction of antioxidative system components^26^.

In *M. crystallinum* the elevated salinity affects the photosynthetic metabolism causing changes in thylakoid organization, expression of photosynthetic proteins and photochemical efficiency^27–29^. We have recently shown that acclimation to salinity for 10 days prevents the decline of linear electron transport observed in control leaves, which already show signs of senescence^30^. We also demonstrated that P700 operates under growth irradiance in a more reduced state in plants acclimated to salinity than in controls, while plastid terminal oxidase (PTOX) is activated only in aging control leaves. These cues suggest a significant changes in the PQ_PA_ pool redox state between controls and salinity-treated plants. It has been postulated earlier that in ice plant, the PQ plays a role in acclimation to salinity, as the C_3_-CAM metabolic shift and transcript abundance of SODs and APX are, at least in part, dependent on redox changes of the photoactive PQ pool^31^. Therefore, in the present work, we aimed at detailed investigating the effect of salinity on the content and redox state of the PQ in *M. crystallinum*.

## Results

### Effect of NaCl treatment on PSII functioning

**Figure 1:**
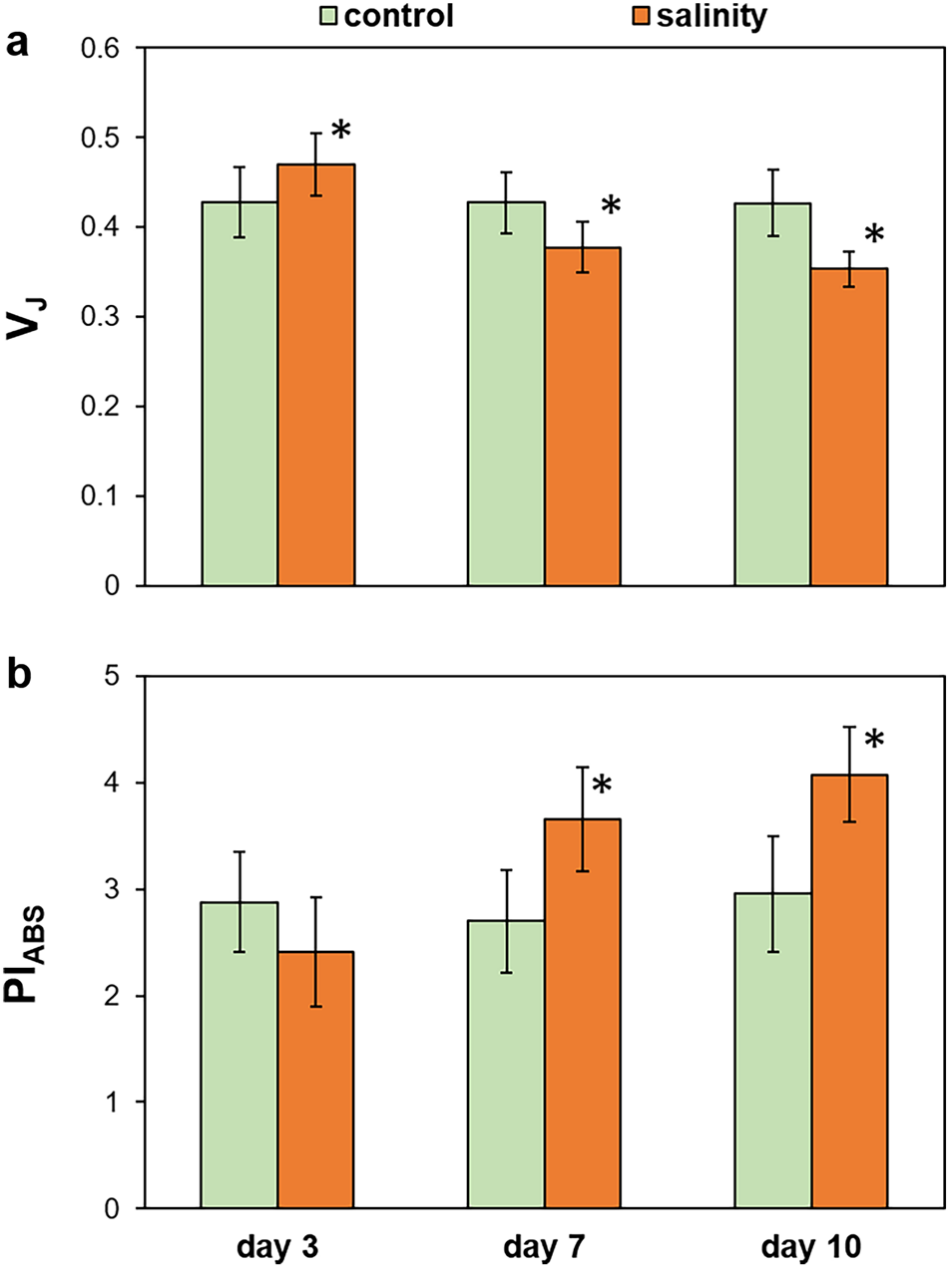
The effect of salinity on the OJIP parameters V_J_ (**a**) and PI_ABS_ (**b**) measured in leaves of *M. crystallinum* plants irrigated with water or with NaCl solution for 3, 7, and 10 days. Values represent mean ± SD (n ≥ 9). Asterisk indicates a significant difference between control and salinity-treated plants at the same time point, as revealed by t test at P ˂ 0.05.

A preliminary insight into the redox state of the PQ pool is given by the non-invasive determination of chlorophyll *a* fluorescence induction curve. The initial photochemical phase of the OJIP transient (the O–J rise) represents a reduction of Q_A_, the primary quinone acceptor, by electrons from PSII^32, 33^. Hence, the relative height of the O–J rise is proportional to the pool of closed PSII reaction centers (RCs). In our experimental model, the relative variable fluorescence at J- step (V_J_) increased after 3 days of NaCl treatment, however prolonged salinity, for 7 and 10 days, decreased V_J_ (Fig. 1a; OJIP transients are shown in Suppl. Fig. S1). This indicates that the salt-acclimated plants have a reduced fraction of closed PSII RCs. The PSII performance index (PI_ABS_) increased in plants after 7 and 10 days of salinity compared with controls (Fig. 1b), which suggests an improved PSII energy conservation efficiency in salt-treated plants.

To verify this conclusion we measured the functioning of PSII in isolated thylakoid membranes. Compared with control plants, the oxygen evolution activity of PSII (from H_2_O to 2,6-dichloro-1,4-benzoquinone, DCBQ) increased in salt-treated plants already on the third day of treatment, and was elevated also under prolonged salinity (on days 7 and 10) (Table 1). Hence, measurements with isolated membranes revealed a more rapid stimulation of PSII activity than those with undamaged leaves. However, the well-watered plants also exhibited enhanced oxygen evolution on day 10 compared with previous time points.

**Table 1:** Oxygen evolution activity (μmol O_2_ mg^−1^ Chl h^−1^) of PSII in isolated thylakoid membranes of *M. crystallinum* irrigated with water or NaCl for 3, 7, and 10 days. O_2_ evolution was determined with the oxygen electrode using DCBQ as electron acceptor. Thylakoids equal to 25 μg ml^−1^ chlorophyll were used (n = 3 ± SD). Means followed by different letters are significantly different at P < 0.05 according to Duncan’s test.

	Day 3	Day 7	Day 10
Control	123.7 ± 5.2 b	130.4 ± 3.4 b	170.0 ± 11.6 a
Salinity	147.6 ± 6.3 c	182.3 ± 4.9 a	199.6 ± 4.5 e

**Figure 2:**
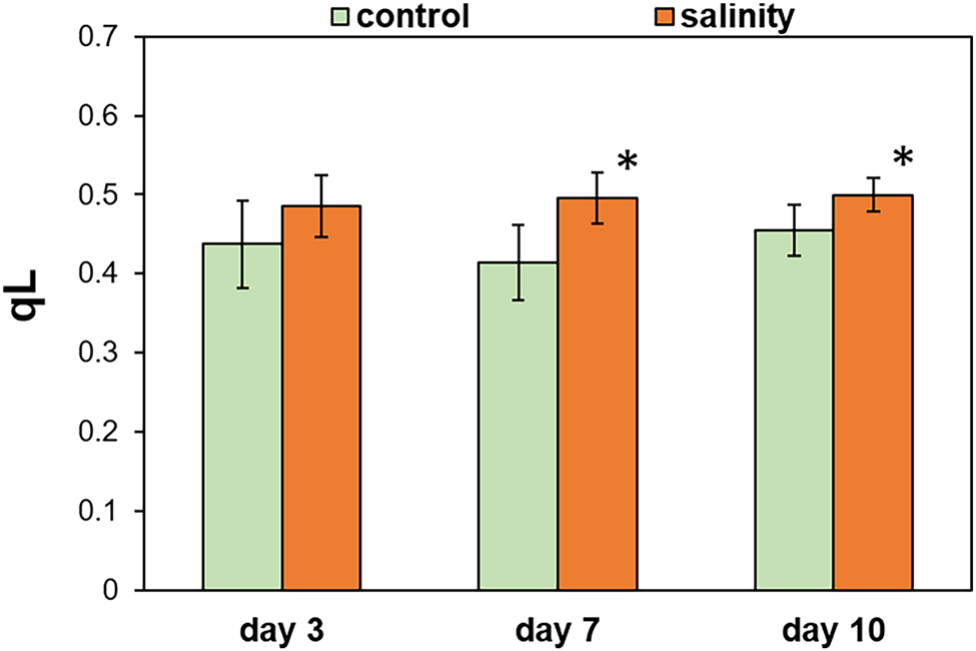
The effect of salinity on the photochemical quenching, qL parameter, measured in leaves of *M. crystallinum* plants irrigated with water or with NaCl solution for 3, 7, and 10 days. Values represent mean ± SD (n ≥ 9). Asterisk indicates a significant difference between control and salinity-treated plants at the same time point, as revealed by t test at P ˂ 0.05.

The conclusion about the enlarged pool of open PSII RCs in salt-treated plants was further supported by the PSII performance under actinic illumination. The proportion of oxidized Q_A_, as envisaged from qL (Fig. 2) and qP (Suppl. Fig. S2), was elevated in NaCl-treated plants on days 7 and 10 compared with the corresponding control. Concomitantly, these results indicate that in *M. crystallinum* prolonged salinity conditions (i.e. a week or more) stimulate the activity of PSII.

The redox state of Q_A_ is influenced by the size of PSII antenna. Such changes can be inferred from variation in chlorophyll *a*/*b* ratio. Our results showed a slight decrease in chlorophyll *a*/*b* ratio in controls after 7 days of treatment, but no differences were observed between well-watered and NaCl-treated plants after 3 and 10 days of salinity (Suppl. Fig. S3a). The amount of LHCII proteins was constant in thylakoids as visualized on Coomassie-stained gels (Suppl. Figure S3b).

### Changes in the size and redox state of the PQ pool

**Figure 3:**
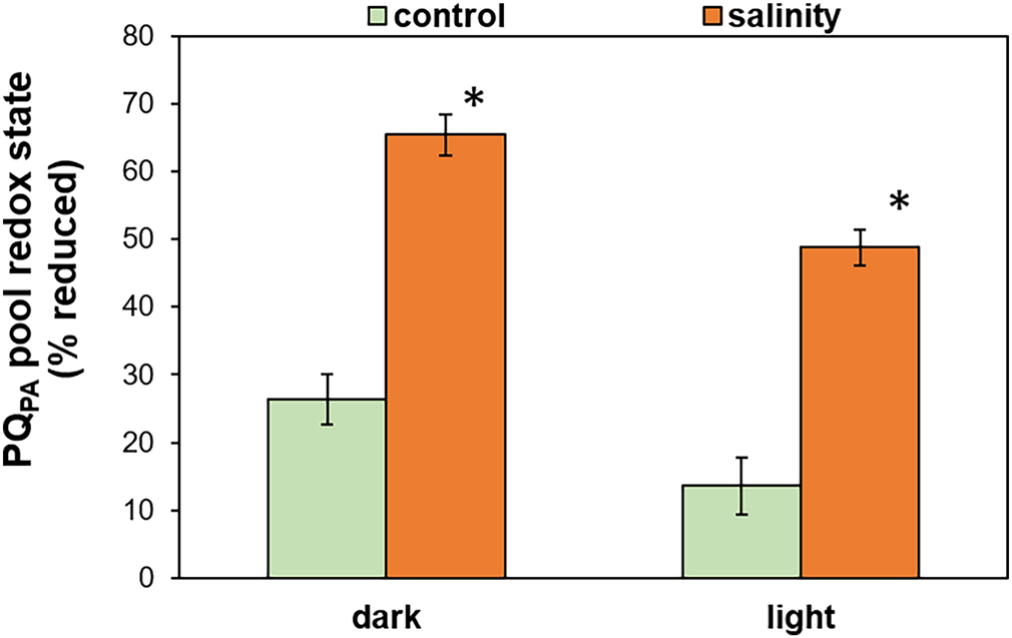
Changes in the redox state of photochemically active PQ fraction (PQ_PA_) in leaves of *M. crystallinum* plants irrigated with water or with NaCl solution for 10 days. Data are means ± SE (n = 3–4). Asterisk indicates a significant difference between control and salinity-treated plants at the same time point, as revealed by t test at P ˂ 0.05.

We analyzed the foliar content of the total PQ pool and its redox state in controls and in plants acclimated to salinity (on day 10 of NaCl treatment). The total PQ amount, in relation to chlorophyll, was not statistically different between analyzed leaves and was 51.6 mol/1000 mol chlorophyll in the control and 46.6 mol/1000 mol chlorophyll in salt-treated plants (Table 2). Next, to assess the size of the fraction involved in PET, we determined the limits of PQ maximal oxidation (in the presence of 3-(3,4-dichlorophenyl)-1,1-dimethylurea, DCMU) and maximal reduction (high light). Based on the obtained data we calculated that the control *M. crystallinum* plants showed 16.9% of PQ in the photochemically active form (PQ_PA_) and 83.1% in the photochemically non-active form (PQ_NP_). Compared with the controls, in plants acclimated to salinity, the proportion of PQ_PA_ increased significantly and amounted to 30.1%, and accordingly, PQ_NP_ was reduced to 69.9% of the total PQ pool. These results suggest that in salt-acclimated plants a larger fraction of open PSII RCs is in contact with a larger pool of photoreducible PQ in the thylakoid membrane. The obtained redox state of PQ_PA_ showed that in control plants this fraction was reduced in 26.3% in darkness, and its reduction level decreased under light conditions and was found to be 13.6% (Fig. 3). The PQ_PA_ in salt-acclimated plants was more reduced compared with control leaves and amounted to 65.4% in the dark and to 48.8% in the light conditions. The redox state of PQ_NP_ was similar in analyzed leaves and in well-watered and NaCl-treated plants this fraction was reduced in about 83%.

**Table 2:** The relative content of total PQ (PQ_tot_, oxidized + reduced) in relation to chlorophyll, size of the photochemically active PQ (PQ_PA_) pool and photochemically non-active PQ (PQ_NP_) pool measured in leaves of *M. crystallinum* plants irrigated with water or with NaCl solution for 10 days. Data are means ± SE (n = 3–4). Asterisk indicates a significant difference between control and salinity-treated plants, as revealed by t test at P ˂ 0.05.

	PQ_tot_/1000 Chl (mol/mol)	PQ_PA_ (% total)	PQ_NP_ (% total)
Control	51.6 ± 2.5	16.9 ± 2.9	83.1 ± 2.9
Salinity	46.6 ± 2.7	30.1 ± 2.1 *	69.9 ± 2.1 *

### Kinetics of PQ_PA_ reduction

**Figure 4:**
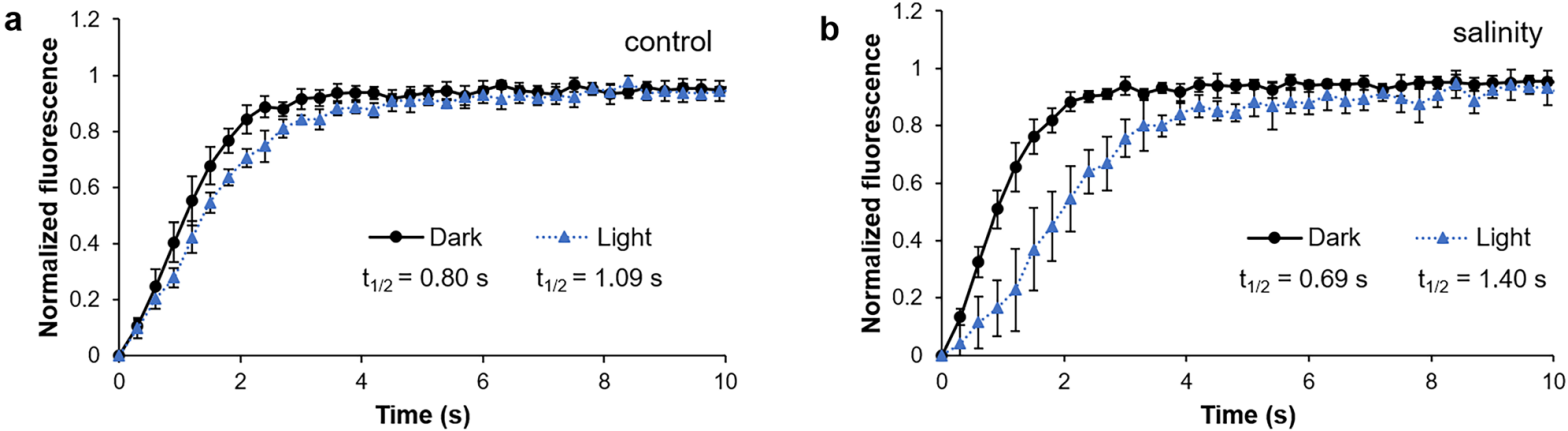
Analyses of PQ reduction via a continuous illumination of limiting intensity in leaves of *M. crystallinum* plants irrigated with water (**a**) or with NaCl solution (**b**) for 10 days. PSII fluorescence induction was monitored in light- or dark-adapted (1 h) leaf-discs infiltrated with DBMIB to block PQ pool oxidation. Traces represent means ± SD (n = 6).

The photoreducible PQ pool can be located in different membrane compartments, grana and stromal lamellae, which affects its reduction rate^34^. To investigate whether the increased amount of PQ_PA_ under prolonged salinity (10 days of treatment) influences the kinetics of its reduction we followed fluorescence induction under continuous illumination and in the presence of 2,5-dibromo-6-isopropyl-3-methyl-1,4-benzoquinone (DBMIB) to avoid PQ_PA_ oxidation by the cytochrome b_6_f. The PQ_PA_ in dark-adapted NaCl-treated plants was more rapidly reduced (t_1/2_ = 0.69 s) compared with control plants (t_1/2_ = 0.80 s) (Fig. 4 a,b). However, in light conditions, the reduction of PQ_PA_ was slower in salt-acclimated plants (t_1/2_ = 1.40 s) than in well-watered ones (t_1/2_ = 1.09 s) which suggests that in NaCl-treated plants larger pool of PQ was available for PSII to reduce.

### Changes in the NADP^+^/NADPH ratio

**Figure 5:**
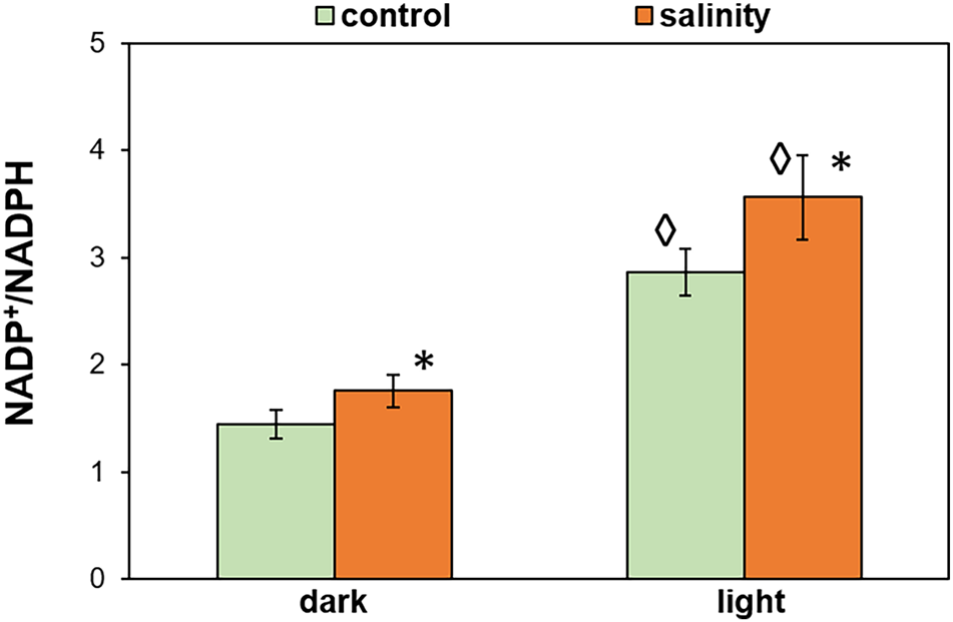
NADP^+^/NADPH ratio in leaves of *M. crystallinum* plants irrigated with water or with NaCl solution for 10 days. Samples for analyses were taken at the end of the nigh (dark) and two hours after the onset of the day (light). Data are means ± SD (n = 3). Asterisk indicates a significant difference between control and salinity-treated plants at the same time point and diamond indicates a statistical significance between the time points of the same treatment, as revealed by t test at P ˂ 0.05.

The electrons can accumulate in the PQ_PA_ pool when NADP^+^ availability is limited, thus we studied the effect of prolonged salinity (10 days of treatment) on the NADP^+^/NADPH ratio in the leaves. In control and NaCl-treated plants, collected just before dawn (dark) and from growth light conditions (light), the NADP^+^/NADPH ratio was higher than 1, meaning that the oxidized form was prevailing (Fig. 5). The imbalance between oxidized and reduced form was higher under light conditions. However, salt-acclimated plants had an increased proportion of NADP^+^ compared with control both in light and in darkness.

## Discussion

### Enlarged pool of open PSII RCs under salinity

In our study the short-term salinity treatment (3 days) had no effect on qL, qP and PI_ABS_. At that time, a transient reduction of the pool of open PSII RCs takes place (V_J_). In contrast, the enlarged pool of open PSII RCs and increased PSII energy conservation efficiency was shown under prolonged salinity (over 7 days). This could result from decreased antennae size or increased amount of active PSII RCs. The first assumption seems unlikely, since no decrease in the relative antennae size was observed based on chlorophyll *a*/*b* and the amount of LHCII proteins.

The isolated thylakoids showed an improved PSII-dependent electron transport (from H_2_O to DCBQ) not only after prolonged but also after short-term NaCl treatment compared with well-watered controls. As generally accepted, DCBQ binds to Q_B_ site of PSII and accepts electrons directly from Q_A_^35^, so this result reflects the maximal rate of PSII photochemistry independent from the PQ pool. Hence, this data strongly support the conclusion about the increased amount of open PSII RCs. This could result from the efficient repair of PSII. Such phenomena under salinity has been previously hypothesized in this species based on higher transcription rate of crucial components of PSII complexes, i.e. psbA and psbD^28^. In other halophytes, the importance of stabilization of PSII under salinity was indicated by enhanced levels of D2 protein in *Suaeda aegyptiaca*^36^ and *Aeluropus lagopoides*^37^ and D1 and CP41 proteins in *Haloxylon salicornicum*^38^.

We also noticed increased oxygen evolution in thylakoids isolated from control leaves, which can be attributed to age. As we showed earlier, well-water control leaves on day 10 show signs of senescence, such as a decline in total chlorophyll content^30^. The decrease in the relative size of the PSII antenna was not observed, thus this result may indicate higher amounts of functional PSII centers in aging controls. However, the non-invasive chlorophyll *a* fluorescence analyses did not reveal any beneficial effect of age on PSII. Therefore, we hypothesize that in vivo PSII efficiency is blocked to a certain extent by the actual redox state of PQ_PA_ while in isolated thylakoids supplied by an artificial electron acceptor, this barrier is removed.

### A highly increased capacity of PQ_PA_ pool for electrons from PSII during daytime in salinity-acclimated plants

HPLC analyses showed a considerable redistribution of the PQ in salt-acclimated plants, as characterized by an increased fraction of PQ pool participating in electron transport at the expense of the PQ_NP_. This might be a natural consequence of the increased pool of open PSII RCs which is in contact with previously inactive PQ fraction. As shown in *A*. *thaliana*, the maintenance of a stable PQ/PSII ratio in thylakoids is important for efficient photosynthesis^39^.

We also revealed that in salinity-acclimated plants, the PQ_PA_ pool becomes more reduced during steady-state photosynthesis in comparison to that in control plants. This is in agreement with the strongly decreased donor side limitation to PSI^28^ which suggests an accumulation of electrons in the intersystem PET. Noteworthy, the contribution of CET to this PQ_PA_ reduction might be excluded on basis of our recent results^30^. However, this observation seems contradictory to the above-mentioned enlarged pool of oxidized PSII RCs in salt-acclimated plants, as it is assumed that Q_A_ in RCs is in equilibrium with the PQ pool^40^. It is worth noting that the assays based on the chlorophyll *a* fluorescence kinetics represent the functional approach describing the probability of electron flow to Q_A_ and revealing the possible limitations. Whereas, the analysis of reduced and oxidized PQ fractions done after the instant leaf freezing illustrates the static situation. Hence, in agreement with the stimulated electron transport from H_2_O to DCBQ, we interpret the data on more reduced PQ_PA_ as a result of intensified electron flux from PSII in salt-acclimated plants.

The physiological role of high PQ_PA_ reduction seems complex. It induces a state transition to state 2^18^ and an indication of this process was previously reported in *M. crystallinum*-CAM plants^41^. It is also known that PQH_2_ is a source of ROS^17^, hence it could play a role in stress signaling under salinity. Using DCMU and DBMIB to block reduction and oxidation of the PQ pool, respectively, it has been demonstrated that its redox state influences the stress responses in *M. crystallinum* through regulation of Cu/Zn-SOD and Fe-SOD expression^31^ and SOD activity^42^. On the other hand, the reduced PQ molecules are also known to be efficient scavengers of the singlet oxygen generated in PSII^43^ and superoxide generated in PSI^16^, therefore providing antioxidant protection.

In contrast to the situation under salinity, in control plants, the proportion of reduced PQ_PA_ (13.6% in the light) appears low as compared with the literature data. In *A. thaliana* grown under low light*,* Kruk and Karpinski^44^ reported a complete reduction of a photochemically active PQ pool, while Szechyńska-Hebda et al.^45^ documented the PQ_PA_ reduction at 40%. In barley, the proportion of reduced PQ_PA_ declined with leaf aging, from 71% in the fully developed first leaf to 44% in the older leaf^46^. The PQH_2_ is re-oxidized by cytochrome b_6_f, but also by PTOX^6^. Indeed, the activation of the latter enzyme was documented earlier in aging well-watered *M. crystallinum* plants^30^, which can explain the untypically high PQ_PA_ oxidation.

An intensified electron flux through the PQ_PA_ pool in salinity-acclimated plants is further supported by the kinetics of PQ_PA_ reduction performed with DBMIB-infiltrated leaves. Here, the slow rate of PQ_PA_ reduction in the light indicates its higher capacity for electrons from PSII. This result might be explained by changes in thylakoid structure. As already mentioned, TEM images have previously shown NaCl-induced thylakoid unstacking and increased gaps between thylakoids in light-acclimated *M*. *crystallinum*^28^. According to the model proposed by Joliot et al.^34^, the domain structure of the plant thylakoid membrane ensures the existence of separate pools of PQ_PA_ in grana and stromal lamellae, therefore thylakoid unstacking in light-acclimated leaves results in a slower reduction of PQ_PA_.

### Nocturnal reduction of PQ_PA_ due to salinity

The low reduction of the PQ_PA_ in control *M*. *crystallinum* plants at night (26%) documented here is in agreement with the data reported earlier for *A*. *thaliana* (24%^44^; 26%^47^). Whereas acclimation to salinity is associated with its high reduction (65%), which is in line with the previous data documented by thermoluminescence^48^. This result is in accordance with the analyses performed with DBMIB-infiltrated leaves, where the reduction rate of PQ_PA_ was faster in dark-adapted salt-treated plants than in controls.

The non-photochemical reduction of PQ_PA_ reflects the metabolic state of the chloroplast and might result from the ATP hydrolysis^49^ or the activity of NAD(P)H dehydrogenase-like complex (NDH)^8, 9^. Both of these electron pathways lead to the accumulation of protons in the thylakoid lumen. Indeed, the non-photochemical quenching, reflecting the development of the proton gradient across thylakoid membranes, has previously been reported in CAM *M*. *crystallinum* at the end of the dark period^28, 29^.

The high dark PQ_PA_ reduction in the salinity-treated plants might be explained by the activation of plastidic starch breakdown in *M. crystallinum*^50, 51^. In CAM plants, an intensive breakdown of starch, glycolysis, and possibly oxidative pentose phosphate pathway are necessary for the nocturnal metabolism associated with fixation of CO_2_ to phosphoenolpyruvate (PEP) and formation of a sufficient pool of malate for the day-time photosynthesis behind closed stomata. In line with that, there are several reports on the nocturnal increase of ATP content in CAM species, *Kalanchoë pinnata*^52^ and ice plant^53^, and reducing power in plastids of *Kalanchoë daigremontiana*^48^. Therefore, it is reasonable to speculate that a part of this nocturnal production of ATP and reducing power can lead to the increased reduction of PQ_PA_.

### The effect of salinity on cellular NADP^+^/NADPH ratio

We demonstrated that salinity shifts the NADP^+^/NADPH ratio towards more NADP^+^, both in dark- and light-adapted plants. This decline of reducing potential in NaCl-treated plants supports earlier data on the disappearance of NDH-dependent CET^30^. Although, this seems not to be a rule for halophytic plants, as Lokhande et al.^54^ reported that the abundance of NADP^+^ level decreased in halophytic *Sesuvium portulacastrum* under salinity. Efficient regeneration of NADP^+^ prevents the over-reduction of chloroplasts and thus protects against elevated ROS production. Therefore, the high NADP^+^/NADPH ratio appears beneficial under salinity.

The described rise in NADP^+^/NADPH ratio suggests that in *M. crystallinum* salt-acclimation is accompanied by increased consumption of reducing power and/or decreased NADPH production compared to controls. Previous studies have shown the lower expression of the ferredoxin-NADP^+^ reductase gene in NaCl-treated plants^55^, which could influence the NADPH yield. However, in ice plant, oxaloacetate reduction^56^, photorespiration^56, 57^, biosynthesis of osmoprotectants^25^ and functioning of an anti-oxidative defence system^58^ may function as strong sinks for reducing power under saline conditions. Therefore a thorough study of NaCl-induced changes in the NADP^+^ turnover is needed.

It is worth noting that generally in this species the redox state of NADP^+^/NADPH couple was more reduced before dawn than after two hours of light-adaptation. This higher nocturnal reducing potential is in agreement with the day/night changes of DHAP/PGA demonstrated earlier in another CAM species, *Kalanchoë daigremontiana*^48^.

## Conclusion

The presented results indicate that in *M*. *crystallinum* the distribution of PQ within the chloroplasts and the redox state of the photochemically active PQ pool are the elements of photosynthetic acclimation to salinity. The performance of linear electron flow at highly reduced PQ might be an indicator of the high salinity resistance in this species.

## Materials and methods

### Plant material and growth conditions

*Mesembryanthemum crystallinum* L. plants (common ice plant, Aizoaceae) were grown from seeds (collection of the Botanical Garden, Darmstadt, Germany) in pots filled with soil in a phytotron chamber under irradiation of 300 µmol photons m^−2^ s^−1^ (white LED) in 12 h photoperiod. The temperature was 25 °C during the day and 18 °C at night, and the relative air humidity was 60–80%. When the fourth pair of leaves was visible plants were irrigated daily for 10 days with 0.4 M NaCl, while the control plants were irrigated with tap water. Such treatment is an established routine for induction of C_3_-CAM shift in this species^28, 59^ and our previous studies confirmed CAM induction, as shown by the increased level of PEP carboxylase after 7 and 10 days of NaCl treatment^30^. All analyses were conducted on the leaves of the third pair.

### Chlorophyll *a* fluorescence

The OJIP transients were recorded after saturating pulse of 3000 μmol m^–2^ s^–1^ from plants dark‐adapted for 25 min, using FluorPen (PSI Photon, Drasov, Czech Republic) according to Strasser et al.^32^. Based on these data the relative variable fluorescence at the J-step (V_J_ = (F_J_ − F_0_)/(F_M_ − F_0_)) was used. The performance index, PI_ABS_ = (RC/ABS) × (φ_Po_/1-φ_Po_) × (ψ_E__o_/1-ψ_E__o_), which accounts for the performance of the photosynthesis apparatus expressed in relation to absorption, was calculated according to Chen et al.^60^. The steady-state measurements of chlorophyll *a* fluorescence were done on plants dark-adapted for 25 min using Multi-Color‐PAM fluorometer (Walz, Effeltrich, Germany). The photochemical energy quenching, qL = (F_m_′ − F_s_)/(F_m_′ − F_o_′) × (F_o_′/F_s_)^61^ was recorded during the induction of photosynthesis under red actinic irradiation of 258 µmol PPFD m^−2^ s^−1^. Chlorophyll fluorescence induction under continuous illumination (light intensity of 6 μmol photons m^−2^ s^−1^) for PQ reduction rate was measured according to Wood et al.^62^ with a Dual PAM 100 (Walz, Effeltrich, Germany) on leaves light- or dark-adapted for 1 h and infiltrated with buffer containing 10 mM HEPES–NaOH pH 7.4, 150 mM sorbitol, 80 μ M DBMIB and 10 mM NH_4_Cl. The half-time of the PQ reduction rate was calculated using GraphPad Prism software (Version 5.03).

### Measurements of PSII oxygen evolution rate

The liquid-phase oxygen electrode (Oxytherm, Hansatech Instruments, UK) was used to estimate the light-dependent oxygen evolution in thylakoids (equivalents to 25 μg chlorophyll). The thylakoids were isolated according to Niewiadomska and Pilarska^30^. PSII-specific electron transport was measured in the presence of 0.25 mM (DCBQ) as an electron acceptor and 10 mM NH_4_Cl was used as an uncoupler^63^.

### The size and redox state of photochemically active and photochemically non-active PQ fractions

The size of photochemically active (PQ_PA_) and photochemically non-active plastoquinone pool (PQ_NP_) and its redox state in *M. crystallinum* leaves after 10 days of NaCl treatment were determined according to Kruk and Karpinski^44^. Briefly, leaf discs (1 cm in diameter) were excised from plants 2 h after the start of the light period and before dawn (12 h of darkness) and were quickly homogenized in a cold mortar with cold ethyl acetate. The extract was transferred to an Eppendorf tube and immediately evaporated to dryness under a stream of nitrogen, and analyzed by high-performance liquid chromatography (HPLC). To obtain a maximal oxidation of PQ_PA_ for reference, the discs were infiltrated with 50 µM DCMU and illuminated at 500 µmol photons m^−2^ s^−1^ for 15 s. To obtain maximal reduction of PQ_PA_ for reference, the discs were exposed to high light (2000 µmol photons m^−2^ s^−1^) for 15 s.

### Estimation of NADP^+^/NADPH ratio

The leaves of *M. crystallinum* after 10 days of treatment were collected 2 h after the start of the light period and after 12 h of darkness (before dawn). A total of 100 mg frozen leaf powder was grounded in a mortar either with 0.2 N HCl or 0.2 N NaOH and heated at 60 °C for 15 min. After centrifuging at 10 000 g for 5 min the supernatant was neutralized and used for NADP^+^/NADPH ratio determination using NADP/NADPH-Glo™ Assay (Promega, Madison, WI, USA) according to manufacturer’s instructions. The luminescence signal was measured by a Synergy2 microplate reader (BioTek, Highland Park, VT, USA).

### Statistical analyses

Student’s t-test or two-way analysis of variance (ANOVA) followed by Duncan’s test, as indicated in figure captions, were used to analyse differences between groups at P < 0.05 using SigmaPlot 12 (Systat Software, Inc, Palo Alto, CA, USA).

## Supplementary Information

Supplementary Information.

## Data Availability

The datasets generated during and/or analyzed during the current study are available from the corresponding author on responsible request. The use of plant parts in the study complies with international, national, and/or institutional guidelines.
