# Supplementary material for: Salinity-induced changes in plastoquinone pool redox state in halophytic Mesembryanthemum crystallinum L
Source: Sci Rep. 2023 Jul 10;13:11160. doi: 10.1038/s41598-023-38194-7 (PMC10333315; doi:10.1038/s41598-023-38194-7)
Supplement: Supplementary file 1 — Supplementary Information. [file 41598_2023_38194_MOESM1_ESM.pdf]

Salinity-induced changes in plastoquinone pool redox state in halophytic *Mesembryanthemum crystallinum* L.

Maria Pilarska[1]*, Ewa Niewiadomska[1], Jerzy Kruk[2]

[1] The F. Górski Institute of Plant Physiology Polish Academy of Sciences, Niezapominajek 21, 30-239 Kraków, Poland
[2] Department of Plant Physiology and Biochemistry, Faculty of Biochemistry, Biophysics and Biotechnology, Jagiellonian University, Gronostajowa 7, 30-387 Kraków, Poland

*Corresponding author: m.pilarska@ifr-pan.edu.pl

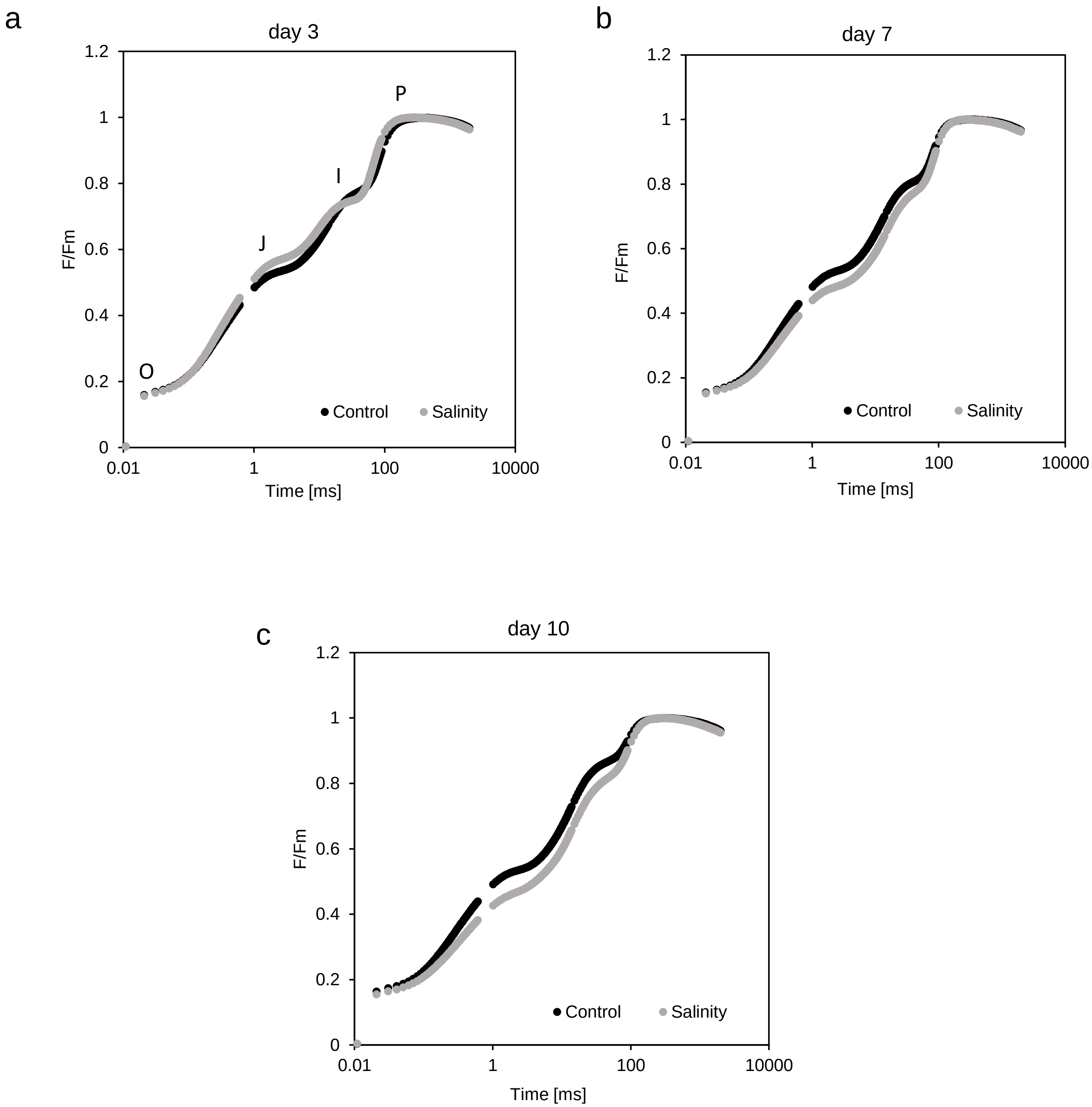

Figure S1. The effect of salinity on the OJIP transients in leaves of *M. crystallinum* plants irrigated with water or with NaCl solution for 3 (a), 7 (b), and 10 days (c). Each curve was based on average data (n ≥ 9).

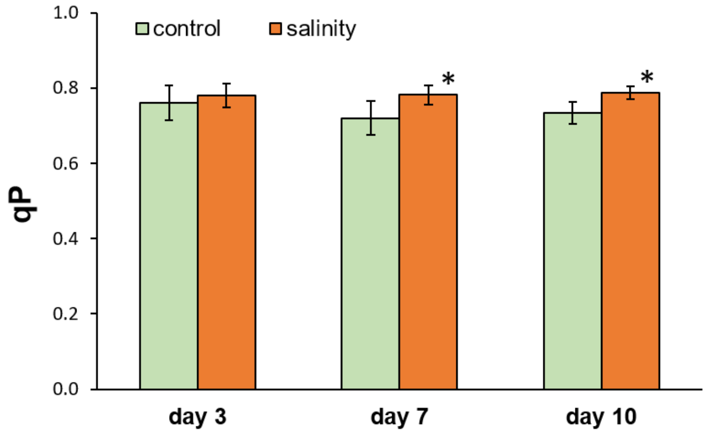

Figure S2. The effect of salinity on the photochemical energy quenching (qP) measured in leaves of *M. crystallinum* plants irrigated with water or with NaCl solution for 3, 7, and 10 days. The qP parameter was followed after the induction of photosynthesis under red actinic irradiation of 258 µmol PPFD $m^{-2}s^{-1}$ ($qP = (F_m'–F_s)/(F_m'– F_o')$) [1]. Values represent mean ± SD ($n \geq 9$). Asterisk indicates a significant difference between control and salinity-treated plants at the same time point, as revealed by t test at $P < 0.05$.

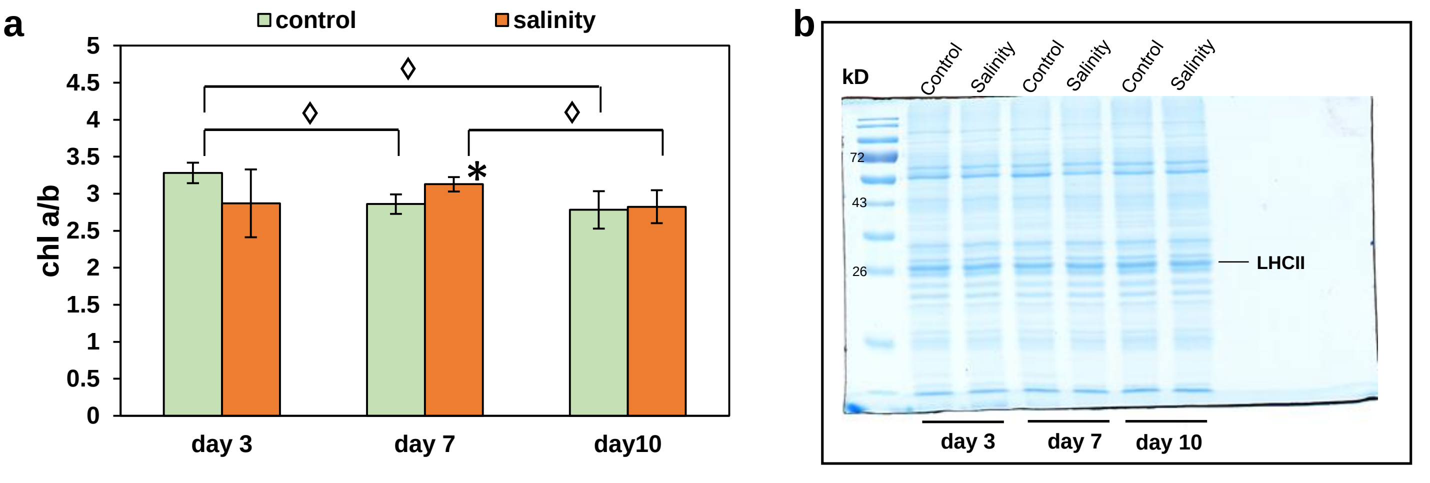

Figure S3. Changes in the chlorophyll *a*/*b* ratio (**a**) and accumulation of LHCII proteins (**b**) in leaves of *M. crystallinum* plants irrigated with water or with NaCl solution for 3, 7, and 10 days. **a** Values represent mean ± SD, n =4. Asterisk indicates a significant difference between control and salinity-treated plants at the same time point and diamond indicates a statistical significance between the time points of the same treatment, as revealed by t test at $P < 0.05$. **b** Thylakoid membranes (1.0 µg of chlorophyll) were subjected to SDS-PAGE and proteins were visualized by Coomassie brilliant blue staining. Gels were scanned using the Epson Perfection V700 Photo scanner (Epson America, Inc., USA). Chlorophyll content determination, thylakoid isolation and SDS-PAGE were performed according to Niewiadomska and Pilarska [2].

References

1. Kramer, D. M., Johnson G., Kiirats O., Edwards G. E. New fluorescence parameters for determination of Q_A redox state and excitation energy fluxes. *Photosynth. Res*. **79**, 209– 218 (2004).
2. Niewiadomska, E., Pilarska, M. Acclimation to salinity in halophytic ice plant prevents a decline of linear electron transport. *Environ. Exp. Bot*. **184**, 104401 (2021).
